# PACAP Enhances Axon Outgrowth in Cultured Hippocampal Neurons to a Comparable Extent as BDNF

**DOI:** 10.1371/journal.pone.0120526

**Published:** 2015-03-25

**Authors:** Katsuya Ogata, Norihito Shintani, Atsuko Hayata-Takano, Toshihiko Kamo, Shintaro Higashi, Kaoru Seiriki, Hisae Momosaki, David Vaudry, Hubert Vaudry, Ludovic Galas, Atsushi Kasai, Kazuki Nagayasu, Takanobu Nakazawa, Ryota Hashimoto, Yukio Ago, Toshio Matsuda, Akemichi Baba, Hitoshi Hashimoto

**Affiliations:** 1 Laboratory of Molecular Neuropharmacology, Graduate School of Pharmaceutical Sciences, Osaka University, Yamadaoka, Suita, Osaka, Japan; 2 Molecular Research Center for Children’s Mental Development, United Graduate School of Child Development, Osaka University, Kanazawa University, Hamamatsu University School of Medicine, Chiba University and University of Fukui, Yamadaoka, Suita, Osaka, Japan; 3 Interdisciplinary Program for Biomedical Sciences, Institute for Academic Initiatives, Osaka University, Yamadaoka, Suita, Osaka, Japan; 4 Neurotrophic Factor and Neuronal Differentiation Team, INSERM U982, DC2N, University of Rouen, Mont-Saint-Aignan, France; 5 PRIMACEN, Cell Imaging Platform of Normandy, Institute for Research and Innovation in Biomedicine (IRIB), University of Rouen, Mont-Saint-Aignan, France; 6 iPS Cell-based Research Project on Brain Neuropharmacology and Toxicology, Graduate School of Pharmaceutical Sciences, Osaka University, Yamadaoka, Suita, Osaka, Japan; 7 Department of Psychiatry, Osaka University Graduate School of Medicine, Yamadaoka, Suita, Osaka, Japan; 8 Laboratory of Medicinal Pharmacology, Graduate School of Pharmaceutical Sciences, Osaka University, Yamadaoka, Suita, Osaka, Japan; 9 Faculty of Pharmaceutical Sciences, Hyogo University of Health Science, Minatojima, Chuo-ku, Kobe, Hyogo, Japan; University of Louisville, UNITED STATES

## Abstract

Pituitary adenylate cyclase-activating polypeptide (PACAP) exerts neurotrophic activities including modulation of synaptic plasticity and memory, hippocampal neurogenesis, and neuroprotection, most of which are shared with brain-derived neurotrophic factor (BDNF). Therefore, the aim of this study was to compare morphological effects of PACAP and BDNF on primary cultured hippocampal neurons. At days *in vitro* (DIV) 3, PACAP increased neurite length and number to similar levels by BDNF, but vasoactive intestinal polypeptide showed much lower effects. In addition, PACAP increased axon, but not dendrite, length, and soma size at DIV 3 similarly to BDNF. The PACAP antagonist PACAP6–38 completely blocked the PACAP-induced increase in axon, but not dendrite, length. Interestingly, the BDNF-induced increase in axon length was also inhibited by PACAP6–38, suggesting a mechanism involving PACAP signaling. K252a, a TrkB receptor inhibitor, inhibited axon outgrowth induced by PACAP and BDNF without affecting dendrite length. These results indicate that in primary cultured hippocampal neurons, PACAP shows morphological actions via its cognate receptor PAC_1_, stimulating neurite length and number, and soma size to a comparable extent as BDNF, and that the increase in total neurite length is ascribed to axon outgrowth.

## Introduction

Pituitary adenylate cyclase-activating polypeptide (PACAP) is a pleiotropic neuropeptide that acts as a neurotransmitter, neuromodulator, and neurotrophic factor via three heptahelical G protein-coupled receptors: a PACAP-preferring (PAC_1_) receptor and two vasoactive intestinal polypeptide (VIP)-shared (VPAC_1_ and VPAC_2_) receptors [1]. PACAP is abundantly expressed in the central nervous system from development to adulthood [2] and is involved in the expression of various higher brain functions including synaptic plasticity and memory [3–5], and stress-related behavioral responses [6–9]. PACAP also exerts neurotrophic and neuroprotective activities [10], such as promotion of neuritogenesis and neurite outgrowth (discussed later), neuroprotection from ischemic insults in the brain [11] and retina [12], and survival of newborn hippocampal neurons generated by enriched environment stimulation *in vivo* [13].

Interestingly, the above mentioned actions of PACAP are mostly shared with neurotrophins such as brain-derived neurotrophic factor (BDNF) [14]. It has been shown that chronic stress dramatically increases PACAP and PAC_1_ receptor, and BDNF and TrkB receptor mRNA expression in the dorsolateral bed nucleus of the stria terminalis (BNST) [6], a nucleus known to mediate chronic stress responses associated with enhanced BNST dendritic branching and volume [15]. This suggests that trophic functions of PACAP and its coordinate effects with chronic stress-induced BNST BDNF and TrkB transcript expression, may underlie maladaptive BNST remodeling and plasticity associated with stress induced behavioral changes [6,16]. Recently, we demonstrated in PACAP-deficient mice that an enriched environment restores behavioral abnormalities [17], and that the survival rate of newly generated hippocampal neurons under enriched rearing decreases while proliferation is normal [13]. Additionally, the increase of BDNF levels in the hippocampus induced by enriched rearing is not affected in PACAP-deficient mice (our unpublished observation). These findings suggest that PACAP signaling is critically involved in neuroplastic changes responsible for environmental stimuli that are at least partly mediated via cytoarchitectural changes, either in cooperation with, or independently of, BDNF signaling.

The neuritogenic activities of PACAP, determined by total neurite length and/or percentage of neurite-bearing cells, are well documented in PC12 [18,19], SH-SY5Y [20], embryonic stem [21], primary cortical precursor [22], cerebellar granule [23], and dorsal root ganglion [24] cells. Recent comprehensive morphological studies in PC12 cells also showed that PACAP increases neurite number per cell, number of branch points per neurite [25], and median cell diameter [26]. However, an inhibitory action of PACAP on increased dendritic length and number elicited by bone morphogenic protein (BMP)-7 was also reported in cultured postganglionic sympathetic neurons [27]. In cultured hippocampal neurons, recent studies show that PACAP increases neurite length during the first 2 days *in vitro* (DIV) [28], and in neurons cultured for 12–14 DIV [29], but an earlier report found that PACAP does not affect the number of dendrites and branches in neurons at 2 DIV [30], suggesting that PACAP exerts complex effects during developmental neuritogenesis. *In vivo*, it has been shown that PACAP-deficient mice exhibit abnormal axonal arborization in the subgranular zone of the dentate gyrus, which is ascribed to elevated expression of stathmin 1 that interacts with tubulin and destabilizes microtubules [31].

Cultured hippocampal neurons are a good model to address sequential development of mature neurons [32]. In these cells, five morphological steps are defined: a lamellipodia extension around the cell body (Stage I); an establishment of several, and apparently identical, processes (Stage II); the extension of one of the processes as an axon (Stage III); the elongation of the remaining processes as dendrites (Stage IV); and finally, the maturation (elongation and branching) of the axon and dendrites (Stage V) [33]. Using this model, BDNF has been shown to exert multiple promotive effects on development and maturation of axons and dendrites [34–36].

In the present study, we aimed to examine the detailed morphological effects of PACAP during development *in vitro*, and compared them with BDNF in primary cultured hippocampal neurons.

## Materials and Methods

### Cell culture and reagent treatment

All animal care and handling procedures were performed in accordance with protocols approved by the Animal Care and Use Committee of the Graduate School of Pharmaceutical Sciences, Osaka University. Primary cultures of hippocampal neurons were prepared as described [37], with minor modifications. Hippocampi were collected from E15–17 fetuses obtained from pregnant mothers (ICR strain; Japan SLC, Kyoto, Japan), incubated with 0.02% EDTA for 15 min at 37°C, and dissociated by repeated trituration with a pipette. Cells were plated in Neurobasal medium (Life Technologies, Carlsbad, CA, USA) supplemented with B27 (2%; Life Technologies), L-glutamine (2 mM), 100 U/ml penicillin, and 0.1 mg/ml streptomycin (all from Nacalai Tesque, Kyoto, Japan), at 2.5 × 10^4^ cells per well in 24-well dishes containing glass coverslips coated with poly-L-lysine. Resulting cultures consisted of 90–95% neurons as determined by microtubule-associated protein 2 (MAP2) immunoreactivity. PACAP (PACAP-38), PACAP6–38, and VIP were purchased from Peptide Institute (Osaka, Japan), human recombinant BDNF was from Peprotech (Rocky Hill, NJ, USA), and K252a was from Sigma-Aldrich (St. Louis, MO, USA). PACAP6–38 and K252a were added 30 min before the addition of the peptides or BDNF.

### Immunocytochemistry

The procedure was essentially as described previously [37]. Briefly, cells were fixed with 4% paraformaldehyde, permeabilized with 0.3% Triton X-100, incubated with a rabbit polyclonal anti-MAP2 antibody (1:200; Millipore Japan, Osaka, Japan) and a mouse monoclonal anti-phospho-neurofilament (pNF) antibody (1:250; Covance Japan, Tokyo, Japan), and with species-specific fluorophore-conjugated secondary antibodies (1:1000; Alexa 488-conjugated anti-rabbit IgG and Alexa 594-conjugated anti-mouse IgG; Molecular Probes, Tokyo, Japan). Fluorescent images were captured using a BIO-REVO BZ-9000 fluorescence microscope (Keyence, Osaka, Japan).

### Morphological analysis

Total neurite length and neurite number per neuron were determined by manual tracing. Axon and dendrite length, soma size, number of primary neurites (which emerge from the soma and often split into more than one neurite segment) per neuron were determined for each individual cell using the BIO-REVO analysis platform (Keyence).

### Statistical analysis

Statistical analyses were performed using Statview (SAS Institute Japan Ltd., Tokyo, Japan), and significant differences determined by one- or two-way ANOVA followed by Tukey—Kramer tests. The threshold for statistical significance was defined as *P* < 0.05.

## Results

### PACAP and BDNF comparably increase total neurite length and number of total and primary neurites

**Fig 1:**
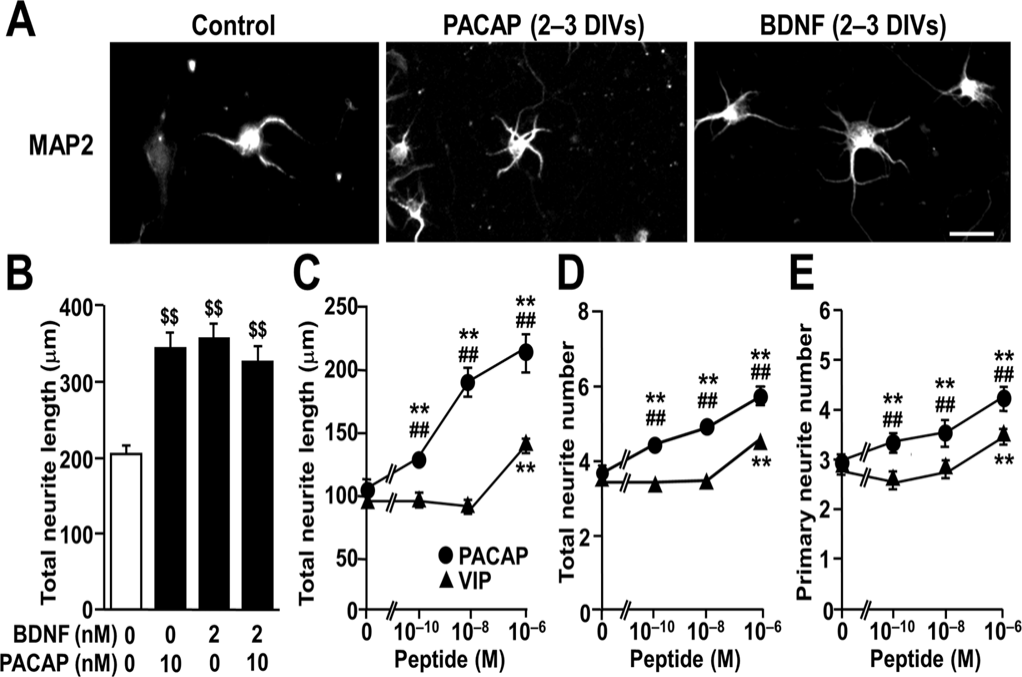
Comparable effects of PACAP and BDNF treatment on total neurite length in cultured hippocampal neurons at DIV 3. Primary hippocampal neurons were cultured with PACAP or BDNF for 2 to 3 DIV and immunostained for MAP2. (A) Representative MAP2-immunostained images of neurons at DIV 3. (B) Total neurite length of cultured hippocampal neurons treated with 10 nM PACAP and/or 2 nM BDNF. (C-E) Dose-dependent effects of PACAP (circles) and VIP (triangles) on total neurite length (C), and number of total (D) and primary (E) neurites. Values represent mean ± SEM of 69–75 neurons from three independent experiments. ^$ $^ *P* < 0.01 vs. control, one-way ANOVA followed by Tukey-Kramer test; **P < 0.01 vs. control, ^##^ *P* < 0.01 vs. identical VIP dose, two-way ANOVA followed by Tukey-Kramer test. Scale bar, 20 μm.

A recent study conducted on primary cultured hippocampal neurons found that exogenous PACAP dose-dependently increases the ratio of neurite length to soma size during the first 2 DIV [28]. In accordance with this, we observed increased total neurite length with PACAP treatment for 2 to 3 DIV in primary cultured hippocampal neurons (Fig. 1A, B). Moreover, quantitative analysis showed that 10^-10^ to 10^-6^ M PACAP dose-dependently increased not only total neurite length, but also total and primary neurite number, while VIP had much lower effects (Fig. 1C-E). A sub-maximal PACAP dose (10 nM; Fig. 1C) increased total neurite length to a similar extent as BDNF (2 nM), but no significant additive effects were observed after co-treatment with PACAP and BDNF (Fig. 1B).

### Time-course analysis on the morphological effects of PACAP

**Fig 2:**
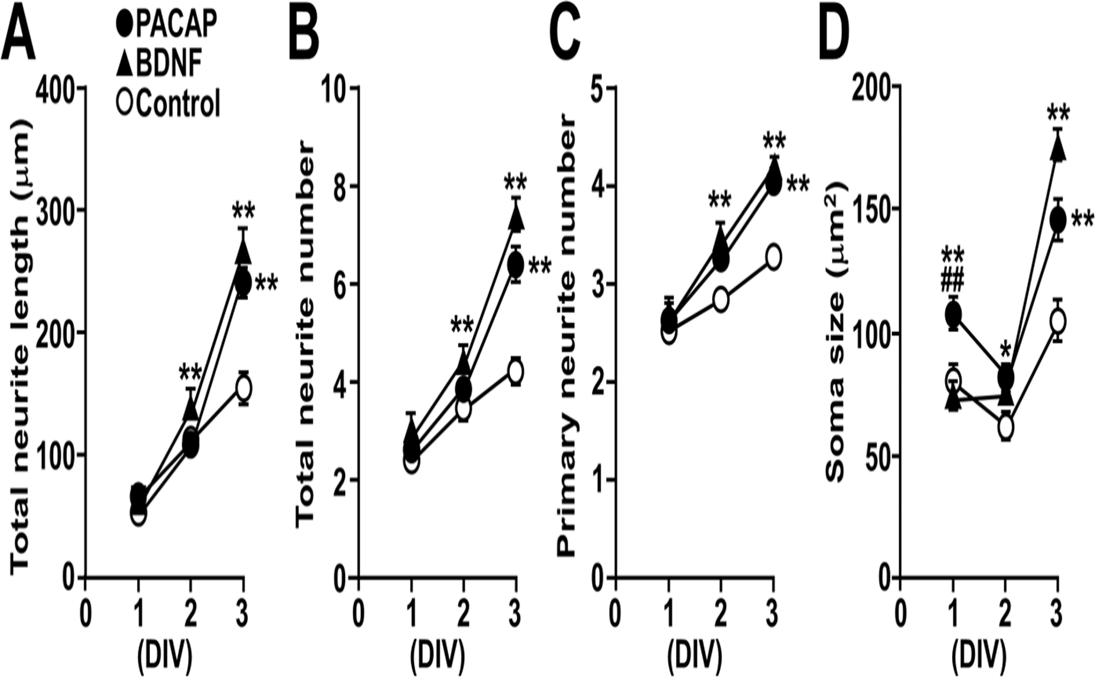
Time course of the morphological effects of PACAP and BDNF on cultured hippocampal neurons during 3 DIV. Primary hippocampal neurons cultured with 10 nM PACAP or 2 nM BDNF were immunostained for MAP2 on DIV 1, 2, and 3. (A-D) Time-dependent effects of PACAP (closed circles), BDNF (closed triangles), and vehicle (open circles) on total neurite length (A), number of total (B) and primary (C) neurites, and soma size (D). Values represent mean ± SEM of 60–75 neurons from three independent experiments. *P < 0.05, **P < 0.01 vs. control at the same DIV, ^##^ *P* < 0.01 vs. BDNF at the same DIV, two-way ANOVA followed by Tukey-Kramer test.

A time-course analysis on the morphological effects of PACAP during early culture period (DIV 1–3) was performed and compared with BDNF (Fig. 2). PACAP increased total neurite length, total and primary neurite number at DIV 3, while BDNF showed significant effects from as early as DIV 2 (Fig. 2A-C). PACAP induced a transient increase in soma size at DIV 1 through 3, while BDNF showed a similar effect at DIV 3 only (Fig. 2D).

### PACAP and BDNF comparably increase axon, but not dendrite, length

**Fig 3:**
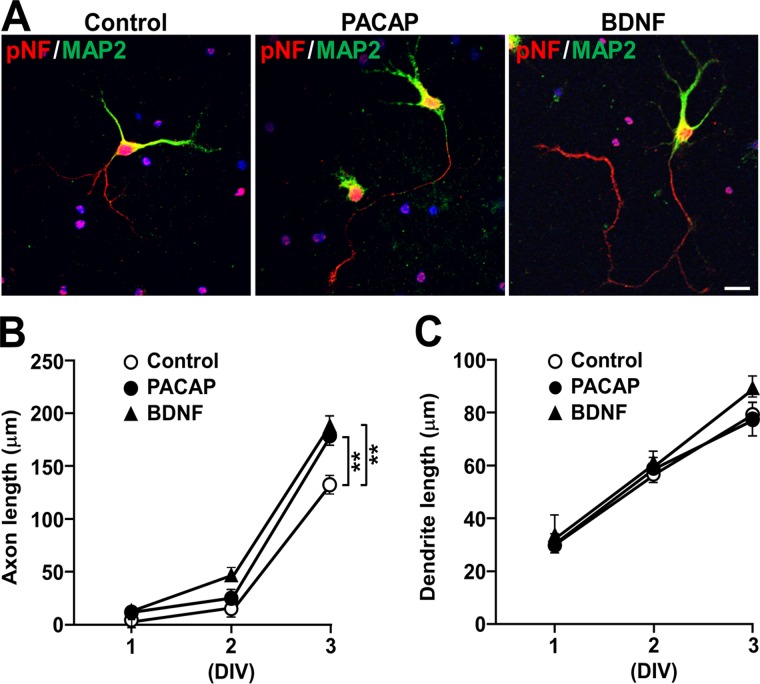
PACAP and BDNF comparably increase axon, but not dendrite, length. Primary hippocampal neurons were cultured with 10 nM PACAP or 2 nM BDNF for 1 to 3 DIV and double-immunostained for pNF and MAP2. (A) Representative pNF- (red) and MAP2- (green) immunostained images of neurons. (B, C) Time-dependent effects of PACAP (closed circles), BDNF (closed triangles), and vehicle (open circles) on axon (B) and dendrite (C) length. Values represent mean ± SEM of 60 neurons from three independent experiments. **P < 0.01, two-way ANOVA followed by Tukey-Kramer test. Scale bar, 20 μm.

Because PACAP increased neurite outgrowth to a comparable extent to BDNF at DIV 3, and as neuronal polarization (axon emergence) is clearly seen around DIV 2 and 3 in rat hippocampal cell cultures [33], we next examined the effect of PACAP on pNF-positive neurites (axons) and MAP2-positive and pNF-negative neurites (dendrites) separately (Fig. 3 and S1 Fig.). At DIV 3, almost all neurons bore a single pNF-immunoreactive axon, together with a few MAP2-immunoreactive neurites (Fig. 3A). Quantitative analysis showed that PACAP (10 nM) significantly increased axon length, comparable to BDNF (2 nM; Fig. 3B). Neither PACAP nor BDNF significantly changed dendrite outgrowth at least during the first 3 DIV (Fig. 3C). Total neurite length (calculated as a total of axonal and dendritic length) was increased in neurons treated with PACAP or BDNF for 3 DIV (both *P* < 0.01 vs. control; control, 214 ± 9; PACAP, 260 ± 10; BDNF, 294 ± 11). These results suggest that PACAP and BDNF elicit a comparable stimulatory effect on neurite length which is ascribed to axon elongation.

### PACAP- and BDNF-enhanced axon outgrowth is blocked by the PACAP antagonist PACAP6–38

**Fig 4:**
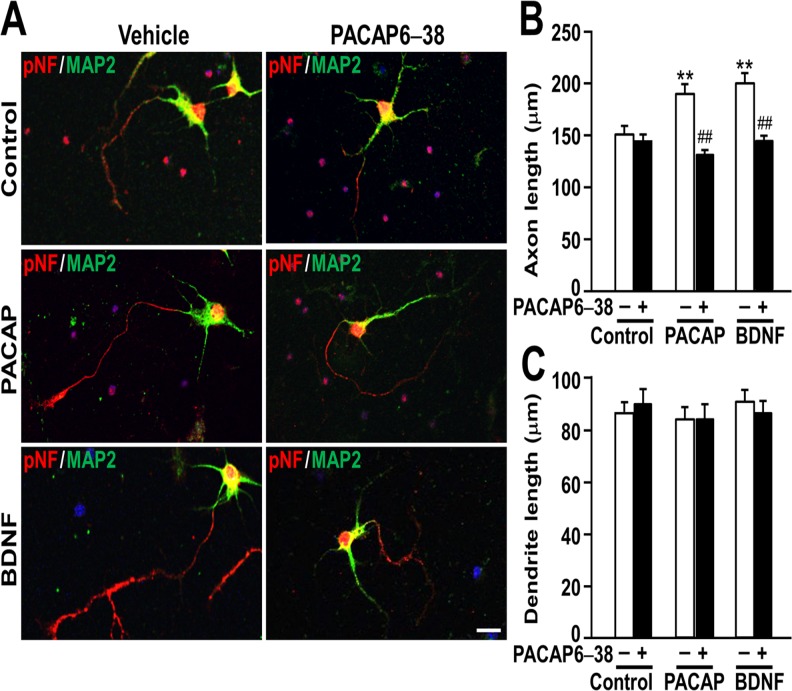
The PACAP antagonist PACAP6–38 blocks the PACAP- and BDNF- induced increase in axon length. Primary hippocampal neurons were cultured with 10 nM PACAP or 2 nM BDNF in the presence or absence of 1 μM PACAP6–38 for 3 DIV and double-immunostained for pNF and MAP2. Representative pNF- (red) and MAP2- (green) immunostained images of neurons (A), axon length (B), and dendrite length (C) were shown. Values represent mean ± SEM of 60 neurons from three independent experiments. **P < 0.01 vs. control, ^##^ *P* < 0.01 vs. without PACAP6–38, two-way ANOVA followed by Tukey-Kramer test. Scale bar, 20 μm.

The observation that VIP had much lower effects on neurite outgrowth than PACAP suggests that the observed effects of PACAP is mediated via PACAP-preferring PAC_1_ receptor but not VIP-shared VPAC_1_ or VPAC_2_ receptor. In agreement with this, the PACAP antagonist PACAP6–38 completely blocked the PACAP-induced increase in axon length, but it showed no effect on axon length of control cultures or dendrite length (Fig. 4). Interestingly, the BDNF-induced increase in axon outgrowth was also inhibited by PACAP6–38, suggesting a mechanism involving PACAP signaling (Fig. 4).

### The TrkB receptor inhibitor K252a strongly inhibited axon, but not dendrite, outgrowth induced by PACAP and BDNF

**Fig 5:**
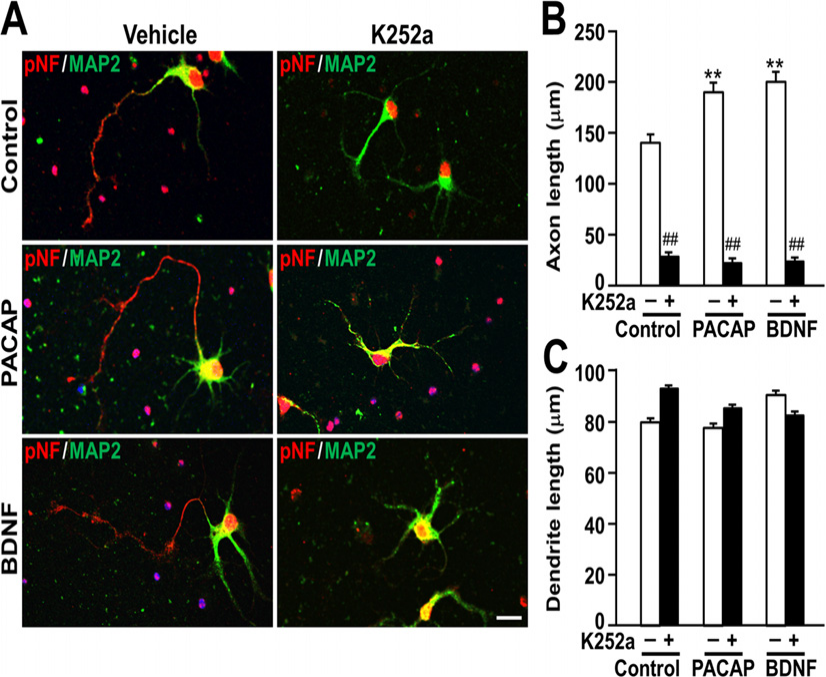
The effect of TrkB receptor inhibitor K252a on neurite outgrowth. Primary hippocampal neurons were cultured with 10 nM PACAP or 2 nM BDNF in the presence or absence of 200 nM K252a for 3 DIV and double-immunostained for pNF and MAP2. Representative pNF- (red) and MAP2- (green) immunostained images of neurons (A), axon length (B), and dendrite length (C) were shown. Values represent mean ± SEM of 60 neurons from three independent experiments. **P < 0.01 vs. control, ^##^ *P* < 0.01 vs. without K252a, two-way ANOVA followed by Tukey-Kramer test. Scale bar, 20 μm.

In order to address if PACAP shows morphogenic effects under inhibition of TrkB, a BDNF receptor, we examined the effect of K252a on neurite outgrowth (Fig. 5). K252a markedly decreased axon length in the neurons treated with PACAP or BDNF (Fig. 5A, B). However, K252a also inhibited axon length of control cultures. In contrast, K252a did not affect dendrite length (Fig. 5A, C).

## Discussion

In the present study, we addressed the morphological effects of PACAP during early *in vitro* development of primary cultured hippocampal neurons, by comparing with BDNF. We found that PACAP increases neurite length, which is specifically due to increased axon, but not dendrite, length, and total and primary neurite number and soma size. These effects of PACAP were mostly comparable to BDNF. The PACAP antagonist PACAP6–38 completely blocked both the PACAP- and BDNF-induced increase in axon length, but not dendrite length, indicating that PACAP shows morphological actions via PAC_1_ receptors and that PACAP signaling might be involved in the BDNF-induced axon outgrowth.

Previous studies in immortalized cell lines have indicated that PACAP has a uniform stimulating effect on various morphological features including total neurite length, total neurite number, extent of branching, and soma size [25,26]. In primary cultured neurons, lack of a stimulatory effect on neurite number was also reported [27,30]. In the present study on primary cultured hippocampal neurons, our observation of a stimulatory effect of PACAP on total neurite outgrowth (total neurite length) is in accordance with recent reports [28,29]. Additionally, we showed that the PACAP-induced increase in total neurite length is ascribed to increased axon, but not dendrite, length, as well as that PACAP increases axon outgrowth, total and primary neurite number, and soma size to a similar extent as BDNF. Henle *et al*. have shown that PACAP does not change the number of dendrites and branches, but reduces elimination of newly formed dendrites and branches caused by NMDA in Stage III hippocampal neurons [30]. It has also been reported that overexpression of full-length TrkB, a BDNF receptor, induces many primary neurites, whereas an alternative Trk receptor isoform (T1) induces net elongation of distal neurites [38]. Although few studies have been performed to address the precise effects of neurotrophic factors on developmental stage-specific regulation of neurite outgrowth, the fact that PACAP enhances axon and neurite outgrowth suggests a distinct property of PACAP on neurite outgrowth.

In the present study, we could conduct morphological analyses of primary cultured hippocampal neurons at DIV 1 through DIV 3 only because pNF-immunoreactive axons elongate and branch vigorously at later DIV and were difficult to be quantified morphologically. To overcome this problem, neurons have to be plated at lower densities and dispersed enough that each neurite can be imaged separately, although different plating densities may affect cell phenotypic properties. Alternatively, transfection of fluorescent proteins with limited efficiency will be a good solution. We plan to address this issue in our future research.

In our preliminary study, we conducted immunostaining only for MAP2 which could address dendrite arborization at later DIV because axons become MAP2 negative after polarization, and examined the effect of PACAP and BDNF on neurons after polarization by treating cultures from DIV 4 to 7. We observed that PACAP and BDNF increased dendrite length to a similar extent (data not shown). This result may suggest that PACAP and BDNF show developmental stage-dependent effects on axons and dendrites, although further study is clearly necessary.

The present observations that K252a inhibited axon outgrowth in the cultures treated with PACAP and BDNF but also in control cultures may not necessarily mean that K252a showed a nonspecific effect because the inhibitor did not affect dendrite length. Previous studies have shown interaction or crosstalk between PACAP and BDNF signaling pathways. In primary cultured hippocampal neurons, PACAP increases BDNF expression via the scaffolding protein, RACK1 [39], and similar to BDNF activation, PACAP induces an increase in phosphorylated TrkB receptors, albeit over a longer time course [40]. It has also been shown in cultured cortical precursor cells that TrkB-immunoreactive cells are increased by PACAP [22], while in mice deficient for the PACAP receptor, PAC_1_, BDNF transcript expression is reduced in the hippocampal CA3 region and dentate gyrus [41]. Dong *et al*. have shown that PACAP induces *BDNF* mRNA expression, which is inhibited by PACAP6–38 or APV, an antagonist for *N*-methyl-D-aspartate receptors (NMDA-R) in cultured rat cortical neurons [42]. Previously, we showed in PC12 cells that PACAP activates Rac1, a small GTPase involved in neurite outgrowth, and acts in synergy with NGF to induce prolonged activation of ERK1/2 and neurite outgrowth [18,43]. Furthermore, we found that NGF and PACAP synergistically enhance PACAP gene transcription, and that the effect of NGF is inhibited by PACAP6–38 [44]. In the present study, we showed that the BDNF-induced increase in axon outgrowth was inhibited by PACAP6–38, suggesting a mechanism involving PACAP signaling in the BDNF action. These findings taken together suggest that mutual interaction between G protein-coupled PAC_1_ receptor and Trk neurotrophin receptor signaling may underlie the robust neurite outgrowth action of PACAP.

The involvement of PACAP in hippocampus-dependent learning and memory is plausible. Mutant mice with either complete or forebrain-specific inactivation of PAC_1_ receptor show a deficit in contextual fear conditioning, a hippocampus-dependent associative learning paradigm, and an impairment of long-term potentiation (LTP) at mossy fiber—CA3 synapses [45]. We previously observed that PAC_1_ receptor exon 2-deficient mice [46] and heterozygous PACAP-deficient mice [47] show an impairment of LTP in the dentate gyrus [4]. It would be intriguing to examine whether intrahippocampal injection of PACAP or a conditionally active PACAP transgene improves memory function.

There is a growing body of evidence implicating PACAP signaling in biological vulnerability to certain psychiatric disorders and stress-related psychopathology. We previously showed that PACAP-deficient mice exhibit remarkable behavioral changes related to psychosis and depression, impairments in memory retention and pre-pulse inhibition [47–52]. We also observed an association between schizophrenia and single nucleotide polymorphisms in the genes for PACAP and the PAC_1_ receptor, as well as an association between the genetic variant of the *PACAP* gene and reduced hippocampal volume and impaired memory performance in schizophrenia [53]. Additionally, a copy number gain in the *PACAP* gene due to a partial trisomy has been shown to cause severe mental retardation [54], and multiplication of the gene for VPAC2, a common VIP and PACAP receptor, is associated with schizophrenia [55]. Furthermore, a sex-specific link between PAC_1_ and post-traumatic stress disorder was demonstrated [56]. As already discussed in the Introduction, Hammack *et al*. have suggested that trophic functions of PACAP and its coordinate effects with chronic stress-induced BDNF and TrkB transcript expression in the BNST may underlie maladaptive BNST remodeling and plasticity associated with stress induced behavioral changes [6,16]. These studies provide convergent evidence for psychiatric implications of the PACAP signaling system; however, the underlying mechanisms remain to be elucidated.

Psychiatric disorders are postulated to be associated with neuroanatomical abnormalities. For example, a reduction in interneuronal neuropil (nerve fibers and branches, and astroglial processes) in the prefrontal cortex has been proposed as a prominent cortical pathological feature of schizophrenia, the so-called “reduced neuropil hypothesis” [57]. Brain imaging studies show not only global anatomical but also functional abnormalities. Most of the neurological disorders associated with alterations in cognition, emotion, and memory loss are often caused by altered synaptic connectivity and plasticity [58]. We previously reported that in primary cultured hippocampal neurons, PACAP regulates an interaction between disrupted-in-schizophrenia 1 (DISC1), a strong candidate gene for schizophrenia, and the central nervous system-specific DISC1-binding zinc finger protein (DBZ) that is involved in neurite extension [59]. Furthermore, it has been shown that PACAP-induced neuritogenesis depends on up-regulation of Egr1 expression [26], a member of the *EGR* gene family involved in regulation of synaptic plasticity, learning, and memory, and implicated in schizophrenia pathogenesis [60]. Thus, a variety of evidence suggests that the morphoregulatory effects of PACAP signaling, either by itself or with Trk neurotrophin signaling, may be implicated in both nervous system development and psychiatric disorders.

## Supporting Information

S1 Fig High magnification images of a primary hippocampal neuron. Primary hippocampal neurons were double-immunostained for pNF (red) and MAP2 (green). Scale bar, 20 μm. The merged image is the same as that of the neuron treated with PACAP only in Fig. 5A.
(PDF)

## References

[1] Vaudry D, Falluel-Morel A, Bourgault S, Basille M, Burel D, Wurtz O, et al Pituitary adenylate cyclase-activating polypeptide and its receptors: 20 years after the discovery. Pharmacol Rev. 2009;61: 283–357. 10.1124/pr.109.001370 19805477

[2] Jaworski DM, Proctor MD. Developmental regulation of pituitary adenylate cyclase-activating polypeptide and PAC(1) receptor mRNA expression in the rat central nervous system. Brain Res Dev Brain Res. 2000;120: 27–39. 10727727 10.1016/s0165-3806(99)00192-3

[3] Roberto M, Scuri R, Brunelli M. Differential effects of PACAP-38 on synaptic responses in rat hippocampal CA1 region. Learn Mem. 2001;8: 265–271. 11584073 10.1101/lm.40501 PMC311380

[4] Matsuyama S, Matsumoto A, Hashimoto H, Shintani N, Baba A. Impaired long-term potentiation in vivo in the dentate gyrus of pituitary adenylate cyclase-activating polypeptide (PACAP) or PACAP type 1 receptor-mutant mice. Neuroreport 2003;14: 2095–2098. 14600504 10.1097/00001756-200311140-00017

[5] MacDonald JF, Jackson MF, Beazely MA. G protein-coupled receptors control NMDARs and metaplasticity in the hippocampus. Biochim Biophys Acta. 2007;1768: 941–951. 17261268 10.1016/j.bbamem.2006.12.006

[6] Hammack SE, Cheung J, Rhodes KM, Schutz KC, Falls WA, Braas KM, et al Chronic stress increases pituitary adenylate cyclase-activating peptide (PACAP) and brain-derived neurotrophic factor (BDNF) mRNA expression in the bed nucleus of the stria terminalis (BNST): roles for PACAP in anxiety-like behavior. Psychoneuroendocrinology 2009;34: 833–843. 10.1016/j.psyneuen.2008.12.013 19181454 PMC2705919

[7] Hashimoto H, Shintani N, Tanida M, Hayata A, Hashimoto R, Baba A. PACAP is implicated in the stress axes. Curr Pharm Des. 2011;17: 985–989. 21524255 10.2174/138161211795589382 PMC3179129

[8] Tsukiyama N, Saida Y, Kakuda M, Shintani N, Hayata A, Morita Y, et al PACAP centrally mediates emotional stress-induced corticosterone responses in mice. Stress 2011;14: 368–375. 10.3109/10253890.2010.544345 21438773 PMC3128825

[9] Smith CB, Eiden LE. Is PACAP the major neurotransmitter for stress transduction at the adrenomedullary synapse? J Mol Neurosci. 2012;48: 403–412. 22610912 10.1007/s12031-012-9749-x PMC4180436

[10] Reglodi D, Kiss P, Szabadfi K, Atlasz T, Gabriel R, Horvath G, et al PACAP is an endogenous protective factor-insights from PACAP-deficient mice. J Mol Neurosci. 2012;48: 482–492. 22528455 10.1007/s12031-012-9762-0

[11] Ohtaki H, Nakamachi T, Dohi K, Aizawa Y, Takaki A, Hodoyama K, et al Pituitary adenylate cyclase-activating polypeptide (PACAP) decreases ischemic neuronal cell death in association with IL-6. Proc Natl Acad Sci U S A 2006;103: 7488–7493. 16651528 10.1073/pnas.0600375103 PMC1464366

[12] Szabadfi K, Atlasz T, Kiss P, Danyadi B, Tamas A, Helyes Z, et al Mice deficient in pituitary adenylate cyclase activating polypeptide (PACAP) are more susceptible to retinal ischemic injury in vivo. Neurotox Res. 2012;21: 41–48. 10.1007/s12640-011-9254-y 21717232

[13] Ago Y, Yoneyama M, Ishihama T, Kataoka S, Kawada K, Tanaka T, et al Role of endogenous pituitary adenylate cyclase-activating polypeptide in adult hippocampal neurogenesis. Neuroscience 2011;172: 554–561. 10.1016/j.neuroscience.2010.10.044 20974227

[14] Park H, Poo MM. Neurotrophin regulation of neural circuit development and function. Nat Rev Neurosci. 2013;14: 7–23. 10.1038/nrn3379 23254191

[15] Pego JM, Morgado P, Pinto LG, Cerqueira JJ, Almeida OF, Sousa N. Dissociation of the morphological correlates of stress-induced anxiety and fear. Eur J Neurosci. 2008;27: 1503–1516. 10.1111/j.1460-9568.2008.06112.x 18336570

[16] Hammack SE, Roman CW, Lezak KR, Kocho-Shellenberg M, Grimmig B, Falls WA, et al Roles for pituitary adenylate cyclase-activating peptide (PACAP) expression and signaling in the bed nucleus of the stria terminalis (BNST) in mediating the behavioral consequences of chronic stress. J Mol Neurosci. 2010;42: 327–340. 10.1007/s12031-010-9364-7 20405238 PMC2955825

[17] Ishihama T, Ago Y, Shintani N, Hashimoto H, Baba A, Takuma K, et al Environmental factors during early developmental period influence psychobehavioral abnormalities in adult PACAP-deficient mice. Behav Brain Res. 2010;209: 274–280. 10.1016/j.bbr.2010.02.009 20144662

[18] Sakai Y, Hashimoto H, Shintani N, Tomimoto S, Tanaka K, Ichibori A, et al Involvement of p38 MAP kinase pathway in the synergistic activation of PACAP mRNA expression by NGF and PACAP in PC12h cells. Biochem Biophys Res Commun. 2001;285: 656–661. 11453643 10.1006/bbrc.2001.5244

[19] Watanabe K, Akimoto Y, Yugi K, Uda S, Chung J, Nakamuta S, et al Latent process genes for cell differentiation are common decoders of neurite extension length. J Cell Sci. 2012;125: 2198–2211. 10.1242/jcs.097709 22344266

[20] Monaghan TK, Mackenzie CJ, Plevin R, Lutz EM. PACAP-38 induces neuronal differentiation of human SH-SY5Y neuroblastoma cells via cAMP-mediated activation of ERK and p38 MAP kinases. J Neurochem. 2008; 104: 74–88. 17995938 10.1111/j.1471-4159.2007.05018.x PMC2230095

[21] Cazillis M, Gonzalez BJ, Billardon C, Lombet A, Fraichard A, Samarut J, et al VIP and PACAP induce selective neuronal differentiation of mouse embryonic stem cells. Eur J Neurosci. 2004;19: 798–808. 15009127 10.1111/j.0953-816x.2004.03138.x

[22] Lu N, DiCicco-Bloom E. Pituitary adenylate cyclase-activating polypeptide is an autocrine inhibitor of mitosis in cultured cortical precursor cells. Proc Natl Acad Sci U S A 1997;94: 3357–3362. 9096398 10.1073/pnas.94.7.3357 PMC20374

[23] Gonzalez BJ, Basille M, Vaudry D, Fournier A, Vaudry H. Pituitary adenylate cyclase-activating polypeptide promotes cell survival and neurite outgrowth in rat cerebellar neuroblasts. Neuroscience 1997;78: 419–430. 9145799 10.1016/s0306-4522(96)00617-3

[24] Nielsen KM, Chaverra M, Hapner SJ, Nelson BR, Todd V, Zigmond RE, et al PACAP promotes sensory neuron differentiation: blockade by neurotrophic factors. Mol Cell Neurosci. 2004;25: 629–641. 15080892 10.1016/j.mcn.2003.12.004

[25] Shi GX, Jin L, Andres DA. Pituitary adenylate cyclase-activating polypeptide 38-mediated Rin activation requires Src and contributes to the regulation of HSP27 signaling during neuronal differentiation. Mol Cell Biol. 2008;28: 4940–4951. 10.1128/MCB.02193-07 18541665 PMC2519709

[26] Ravni A, Vaudry D, Gerdin MJ, Eiden MV, Falluel-Morel A, Gonzalez BJ, et al A cAMP-dependent, protein kinase A-independent signaling pathway mediating neuritogenesis through Egr1 in PC12 cells. Mol Pharmacol. 2008;73: 1688–1708. 10.1124/mol.107.044792 18362103 PMC4188547

[27] Drahushuk K, Connell TD, Higgins D. Pituitary adenylate cyclase-activating polypeptide and vasoactive intestinal peptide inhibit dendritic growth in cultured sympathetic neurons. J Neurosci. 2002;22: 6560–6569. 12151535 10.1523/JNEUROSCI.22-15-06560.2002 PMC6758139

[28] Kambe Y, Miyata A. Role of mitochondrial activation in PACAP dependent neurite outgrowth. J Mol Neurosci. 2012;48: 550–557. 22460784 10.1007/s12031-012-9754-0

[29] Lazarovici P, Cohen G, Arien-Zakay H, Chen J, Zhang C, Chopp M, et al Multimodal neuroprotection induced by PACAP38 in oxygen-glucose deprivation and middle cerebral artery occlusion stroke models. J Mol Neurosci. 2012;48: 526–540. 22678884 10.1007/s12031-012-9818-1 PMC3955207

[30] Henle F, Fischer C, Meyer DK, Leemhuis J. Vasoactive intestinal peptide and PACAP38 control N-methyl-D-aspartic acid-induced dendrite motility by modifying the activities of Rho GTPases and phosphatidylinositol 3-kinases. J Biol Chem. 2006;281: 24955–24969. 16803895 10.1074/jbc.M604114200

[31] Yamada K, Matsuzaki S, Hattori T, Kuwahara R, Taniguchi M, Hashimoto H, et al Increased stathmin1 expression in the dentate gyrus of mice causes abnormal axonal arborizations. PLoS One 2010;5: e8596 10.1371/journal.pone.0008596 20062533 PMC2797614

[32] Caceres A, Ye B, Dotti CG. Neuronal polarity: demarcation, growth and commitment. Curr Opin Cell Biol. 2012;24: 547–553. 10.1016/j.ceb.2012.05.011 22726583 PMC3425660

[33] Dotti CG, Sullivan CA, Banker GA. The establishment of polarity by hippocampal neurons in culture. J Neurosci. 1988;8: 1454–1468. 3282038 10.1523/JNEUROSCI.08-04-01454.1988 PMC6569279

[34] Patel MN, McNamara JO. Selective enhancement of axonal branching of cultured dentate gyrus neurons by neurotrophic factors. Neuroscience 1995;69: 763–770. 8596646 10.1016/0306-4522(95)00281-m

[35] Labelle C, Leclerc N. Exogenous BDNF, NT-3 and NT-4 differentially regulate neurite outgrowth in cultured hippocampal neurons. Brain Res Dev Brain Res. 2000; 123: 1–11. 11020545 10.1016/s0165-3806(00)00069-9

[36] Ji Y, Pang PT, Feng L, Lu B. Cyclic AMP controls BDNF-induced TrkB phosphorylation and dendritic spine formation in mature hippocampal neurons. Nat Neurosci. 2005;8: 164–172. 15665879 10.1038/nn1381

[37] Tajiri M, Hayata-Takano A, Seiriki K, Ogata K, Hazama K, Shintani N, et al Serotonin 5-HT(7) receptor blockade reverses behavioral abnormalities in PACAP-deficient mice and receptor activation promotes neurite extension in primary embryonic hippocampal neurons: therapeutic implications for psychiatric disorders. J Mol Neurosci. 2012;48: 473–481. 22843252 10.1007/s12031-012-9861-y

[38] Yacoubian TA, Lo DC. Truncated and full-length TrkB receptors regulate distinct modes of dendritic growth. Nat Neurosci. 2000;3: 342–349. 10725923 10.1038/73911

[39] Yaka R, He DY, Phamluong K, Ron D. Pituitary adenylate cyclase-activating polypeptide (PACAP(1–38)) enhances N-methyl-D-aspartate receptor function and brain-derived neurotrophic factor expression via RACK1. J Biol Chem. 2003;278: 9630–9638. 12524444 10.1074/jbc.M209141200

[40] Lee FS, Rajagopal R, Kim AH, Chang PC, Chao MV. Activation of Trk neurotrophin receptor signaling by pituitary adenylate cyclase-activating polypeptides. J Biol Chem. 2002;277: 9096–9102. 11784714 10.1074/jbc.M107421200

[41] Zink M, Otto C, Zorner B, Zacher C, Schutz G, Henn FA, et al Reduced expression of brain-derived neurotrophic factor in mice deficient for pituitary adenylate cyclase activating polypeptide type-I-receptor. Neurosci Lett. 2004;360: 106–108. 15082190 10.1016/j.neulet.2004.01.030

[42] Dong YX, Fukuchi M, Inoue M, Takasaki I, Tabuchi A, Wu CF, et al Pituitary adenylate cyclase-activating polypeptide (PACAP) is an upstream regulator of prodynorphin mRNA expression in neurons. Neurosci Lett. 2010;484: 174–177. 10.1016/j.neulet.2010.08.044 20728507

[43] Sakai Y, Hashimoto H, Shintani N, Katoh H, Negishi M, Kawaguchi C, et al PACAP activates Rac1 and synergizes with NGF to activate ERK1/2, thereby inducing neurite outgrowth in PC12 cells. Brain Res Mol Brain Res. 2004;123: 18–26. 15046862 10.1016/j.molbrainres.2003.12.013

[44] Hashimoto H, Hagihara N, Koga K, Yamamoto K, Shintani N, Tomimoto S, et al Synergistic induction of pituitary adenylate cyclase-activating polypeptide (PACAP) gene expression by nerve growth factor and PACAP in PC12 cells. J Neurochem. 2000;74: 501–507. 10646500 10.1046/j.1471-4159.2000.740501.x

[45] Otto C, Kovalchuk Y, Wolfer DP, Gass P, Martin M, Zuschratter W, et al Impairment of mossy fiber long-term potentiation and associative learning in pituitary adenylate cyclase activating polypeptide type I receptor-deficient mice. J Neurosci. 2001;21: 5520–5527. 11466423 10.1523/JNEUROSCI.21-15-05520.2001 PMC6762677

[46] Hashimoto H, Shintani N, Nishino A, Okabe M, Ikawa M, Matsuyama S, et al Mice with markedly reduced PACAP (PAC(1)) receptor expression by targeted deletion of the signal peptide. J Neurochem. 2000;75: 1810–1817. 11032869 10.1046/j.1471-4159.2000.0751810.x

[47] Hashimoto H, Shintani N, Tanaka K, Mori W, Hirose M, Matsuda T, et al Altered psychomotor behaviors in mice lacking pituitary adenylate cyclase-activating polypeptide (PACAP). Proc Natl Acad Sci U S A 2001;98: 13355–13360. 11687615 10.1073/pnas.231094498 PMC60875

[48] Hashimoto H, Hashimoto R, Shintani N, Tanaka K, Yamamoto A, Hatanaka M, et al Depression-like behavior in the forced swimming test in PACAP-deficient mice: amelioration by the atypical antipsychotic risperidone. J Neurochem. 2009;110: 595–602. 10.1111/j.1471-4159.2009.06168.x 19457081

[49] Hazama K, Hayata-Takano A, Uetsuki K, Kasai A, Encho N, Shintani N, et al Increased Behavioral and Neuronal Responses to a Hallucinogenic Drug in PACAP Heterozygous Mutant Mice. PLoS One 2014; 9: e89153 10.1371/journal.pone.0089153 24586556 PMC3930680

[50] Tanaka K, Shintani N, Hashimoto H, Kawagishi N, Ago Y, Matsuda T, et al Psychostimulant-induced attenuation of hyperactivity and prepulse inhibition deficits in Adcyap1-deficient mice. J Neurosci. 2006;26: 5091–5097. 16687500 10.1523/JNEUROSCI.4376-05.2006 PMC6674244

[51] Gaszner B, Kormos V, Kozicz T, Hashimoto H, Reglodi D, Helyes Z. The behavioral phenotype of pituitary adenylate-cyclase activating polypeptide-deficient mice in anxiety and depression tests is accompanied by blunted c-Fos expression in the bed nucleus of the stria terminalis, central projecting Edinger-Westphal nucleus, ventral lateral septum, and dorsal raphe nucleus. Neuroscience 2012;202: 283–299. 10.1016/j.neuroscience.2011.11.046 22178610

[52] Hattori S, Takao K, Tanda K, Toyama K, Shintani N, Baba A, et al Comprehensive behavioral analysis of pituitary adenylate cyclase-activating polypeptide (PACAP) knockout mice. Front Behav Neurosci. 2012;6: 58 10.3389/fnbeh.2012.00058 23060763 PMC3462416

[53] Hashimoto R, Hashimoto H, Shintani N, Chiba S, Hattori S, Okada T, et al Pituitary adenylate cyclase-activating polypeptide is associated with schizophrenia. Mol Psychiatry 2007;12: 1026–1032. 17387318 10.1038/sj.mp.4001982

[54] Freson K, Hashimoto H, Thys C, Wittevrongel C, Danloy S, Morita Y, et al The pituitary adenylate cyclase-activating polypeptide is a physiological inhibitor of platelet activation. J Clin Invest. 2004;113: 905–912. 15067323 10.1172/JCI19252 PMC362113

[55] Vacic V, McCarthy S, Malhotra D, Murray F, Chou HH, Peoples A, et al Duplications of the neuropeptide receptor gene VIPR2 confer significant risk for schizophrenia. Nature 2011;471: 499–503. 10.1038/nature09884 21346763 PMC3351382

[56] Ressler KJ, Mercer KB, Bradley B, Jovanovic T, Mahan A, Kerley K, et al Post-traumatic stress disorder is associated with PACAP and the PAC1 receptor. Nature 2011;470: 492–497. 10.1038/nature09856 21350482 PMC3046811

[57] Selemon LD, Goldman-Rakic PS. The reduced neuropil hypothesis: a circuit based model of schizophrenia. Biol Psychiatry 1999;45: 17–25. 9894571 10.1016/s0006-3223(98)00281-9

[58] Sala C, Segal M. Dendritic spines: the locus of structural and functional plasticity. Physiol Rev. 2014;94: 141–188. 10.1152/physrev.00012.2013 24382885

[59] Hattori T, Baba K, Matsuzaki S, Honda A, Miyoshi K, Inoue K, et al A novel DISC1-interacting partner DISC1-Binding Zinc-finger protein: implication in the modulation of DISC1-dependent neurite outgrowth. Mol Psychiatry 2007;12: 398–407. 17389905 10.1038/sj.mp.4001945

[60] Cheng MC, Chuang YA, Lu CL, Chen YJ, Luu SU, Li JM, et al Genetic and functional analyses of early growth response (EGR) family genes in schizophrenia. Prog Neuropsychopharmacol Biol Psychiatry 2012;39: 149–155. 10.1016/j.pnpbp.2012.06.004 22691714

