# Supplementary figures and images for: PACAP Enhances Axon Outgrowth in Cultured Hippocampal Neurons to a Comparable Extent as BDNF
Source: PLoS One. 2015 Mar 25;10(3):e0120526. doi: 10.1371/journal.pone.0120526 (PMC4373823; doi:10.1371/journal.pone.0120526)

**Figure S1. High magnification images of a primary hippocampal neuron**

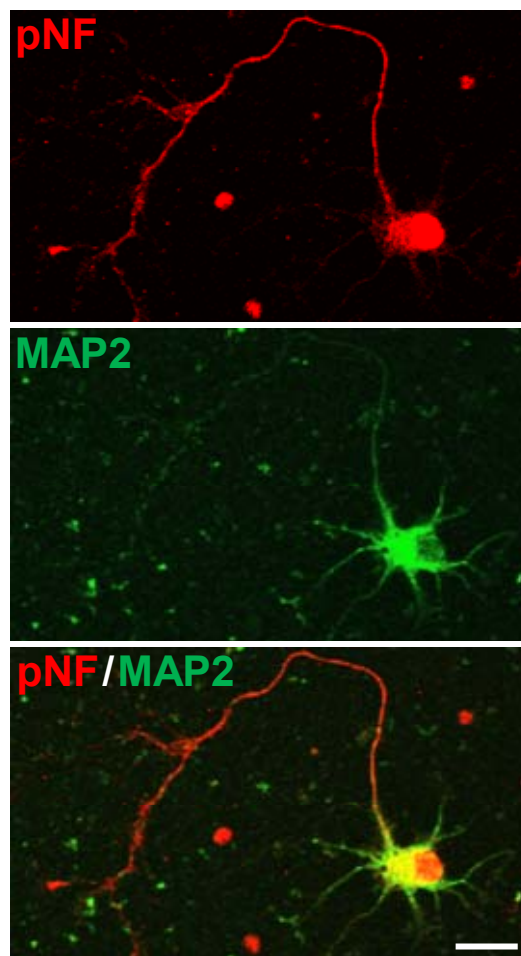

Supplement: S1 Fig — Primary hippocampal neurons were double-immunostained for pNF (red) and MAP2 (green). Scale bar, 20 μm. The merged image is the same as that of the neuron treated with PACAP only in Fig. 5A. (PDF) [file pone.0120526.s001.pdf]
